# Supplementary material for: Manipulating Li2S2/Li2S mixed discharge products of all-solid-state lithium sulfur batteries for improved cycle life
Source: Nat Commun. 2023 Oct 12;14:6404. doi: 10.1038/s41467-023-42109-5 (PMC10570351; doi:10.1038/s41467-023-42109-5)
Supplement: Supplementary file 1 — Supplementary Information [file 41467_2023_42109_MOESM1_ESM.pdf]

Supplementary Information

Manipulating Li_2S_2/Li_2S Mixed Discharge Products of All-Solid-State Lithium Sulfur Batteries for Improved Cycle Life

Jung Tae Kim[1], Adwitiya Rao[2], Heng-Yong Nie[3,4], Yang Hu[1], Weihan Li[1], Feipeng Zhao[1], Sixu Deng[1], Xiaoge Hao[1], Jiamin Fu[1], Jing Luo[1], Hui Duan[1], Chandra Veer Singh[2,*], Changhong Wang[5,*], and Xueliang Sun[1,5*]

[1] Department of Mechanical and Materials Engineering, University of Western Ontario, 1151 Richmond St, London, Ontario, N6A 3K7, Canada.

[2] Department of Materials Science and Engineering, University of Toronto, Ontario, M5S 3E4 Canada.

[3] Surface Science Western, University of Western Ontario, 999 Collip Circle, London, Ontario, N6A 3K7, Canada.

[4] Department of Physics and Astronomy, University of Western Ontario, 1151 Richmond St, London, Ontario, N6A 3K7, Canada.

[5] Eastern Institute for Advanced Study, Ningbo 315201, China

* Corresponding email: chandraveer.singh@utoronto.ca, changhongwang@eias.ac.cn and xsun9@uwo.ca

Sulfur content (wt %)	Solid-state electrolyte content (wt %)	Carbon additive content (wt %)	Reference
40	40	20	Sakuda et al.[1]
40	50	10	Sun et al.[2]
30	60	10	Han et al.[3]
20	40	20	Hou et al.[4]
30	50	20	Zhang et al.[5]
30	50	20	Yao et al.[6]
20	40	20	Zhu et al.[7]
24	60	16	Wang et al.[8]
25	60	15	Zhang et al.[9]

Supplementary Table 1. Summary of the sulfur, solid-state electrolyte, and carbon additive ratio used in recent studies to fabricate the composite electrode of ASSLSBs.

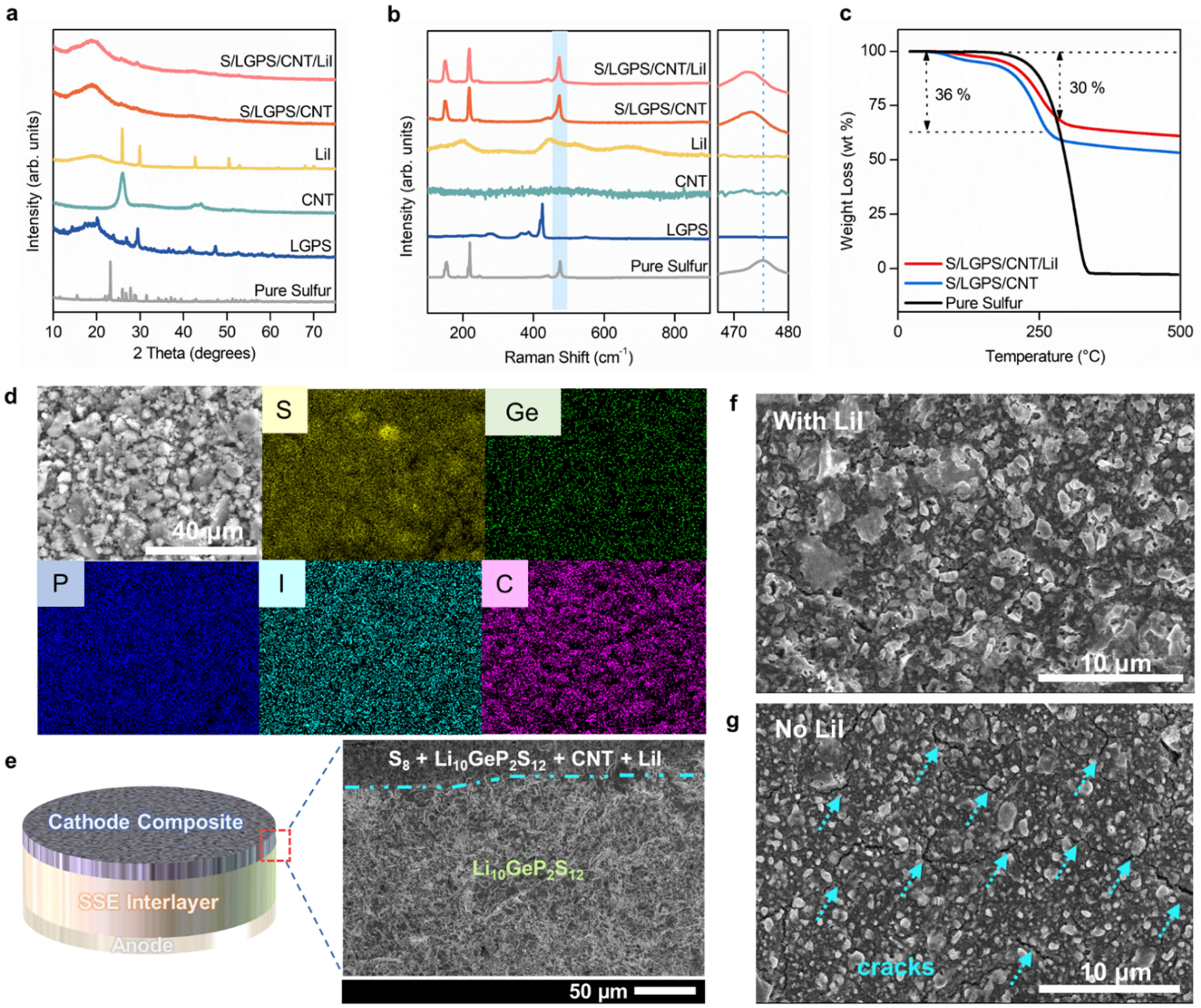

Supplementary Figure 1. Structural characterization of S composite electrodes with and without LiI. (**a**) XRD patterns of S composite electrodes with and without LiI compared against S, LGPS, CNT, and LiI. (**b**) Raman spectra of S composite electrodes with and without LiI compared against S, LGPS, CNT, and LiI. (**c**) TGA curves of S composite electrodes with and without LiI against S. (**d**) SEM image of the LiI-incorporated S composite electrode after the ball-milling process with corresponding EDX mapping of S, Ge, P, I, and C elements. (**e**) Schematic illustration and cross-sectional SEM image of the interface between the LiI-incorporated S composite electrode and SSE

interlayer pelletized at 350 MPa. (**f**) SEM image (top view) of the LiI-incorporated S composite electrode pelletized at 350 MPa. (**g**) SEM image (top view) of the S composite electrode without LiI pelletized at 350 MPa.

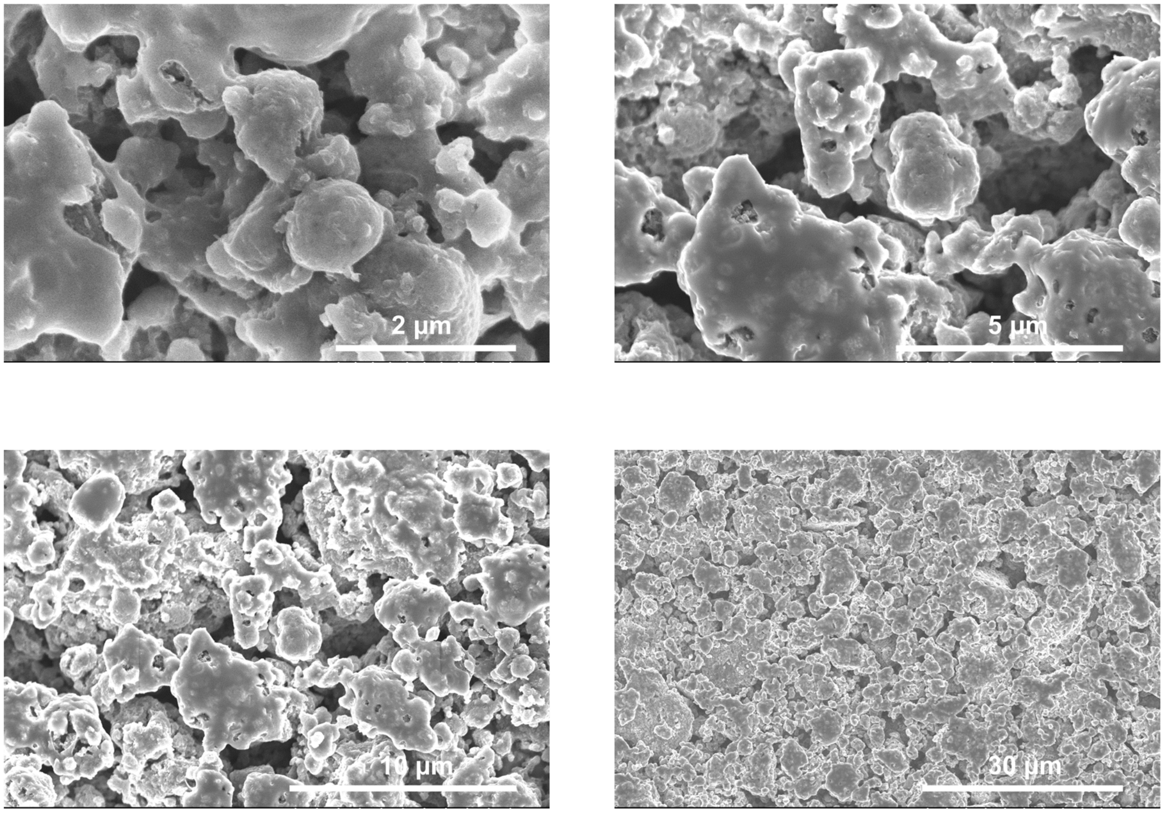

Supplementary Figure 2. SEM images of the LiI-incorporated S composite electrode after ball-milling under different magnfication.

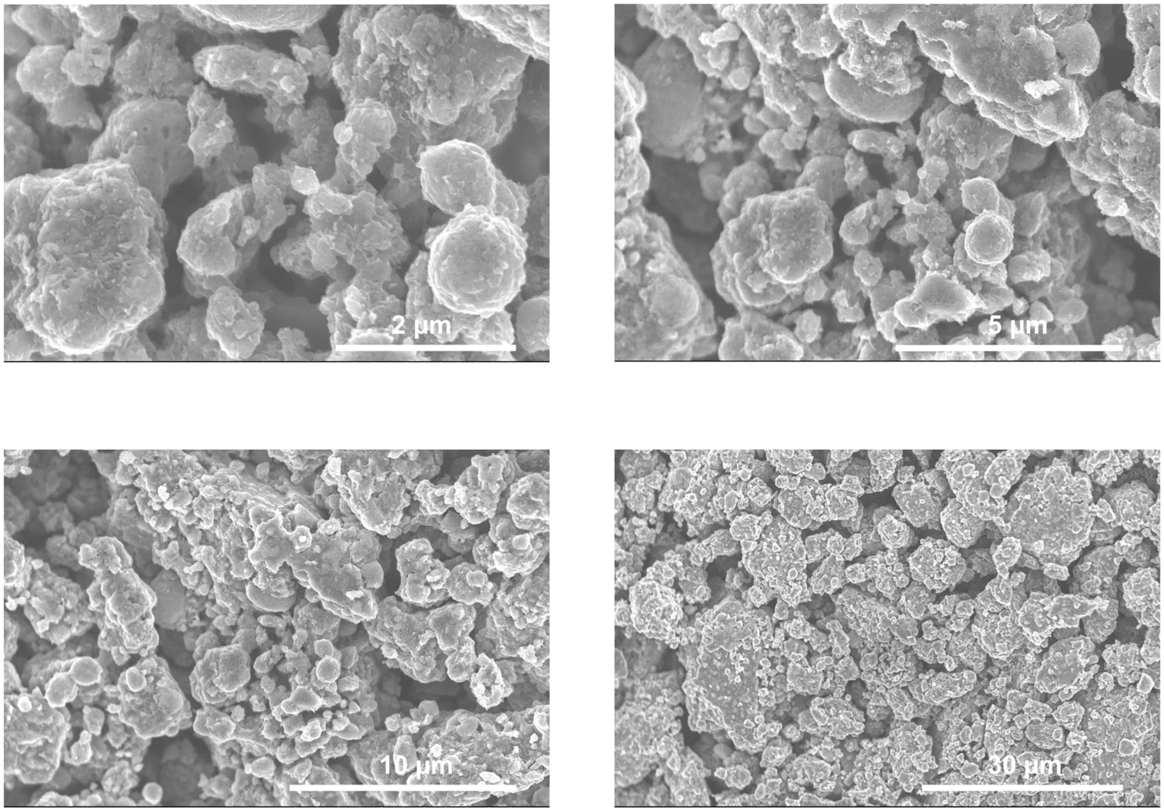

Supplementary Figure 3. SEM images of the S composite electrode without LiI after ball-milling under different magnfication.

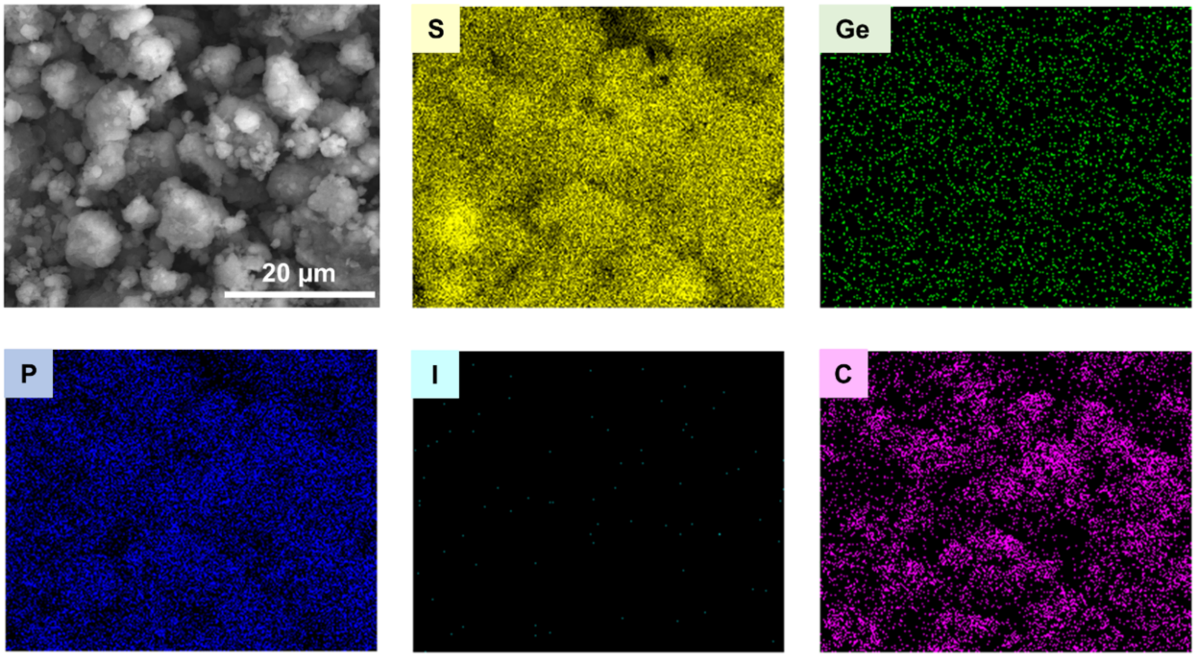

Supplementary Figure 4. SEM image and EDX mapping of the S composite electrode without LiI after ball-milling.

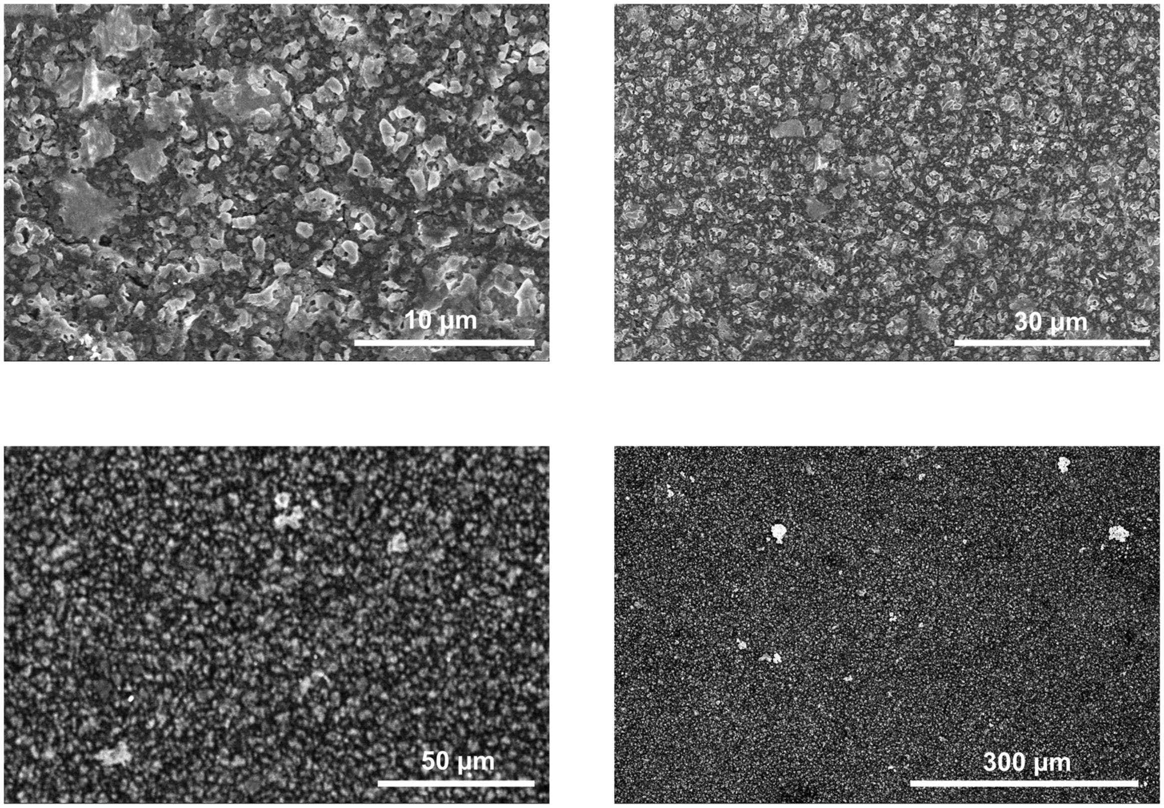

Supplementary Figure 5. Top view SEM images of the LiI-incorporated S composite electrode pelletized at 350MPa under different magnification.

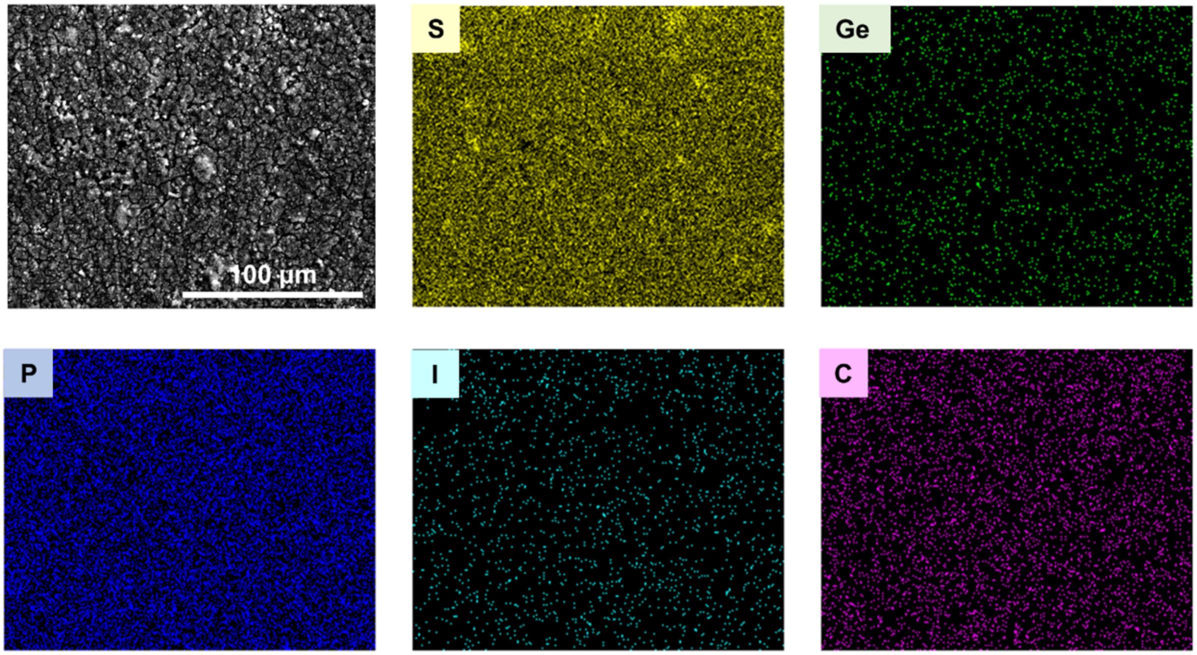

Supplementary Figure 6. Top view SEM image and EDX mapping of the LiI-incorporated S composite electrode pelletized at 350MPa.

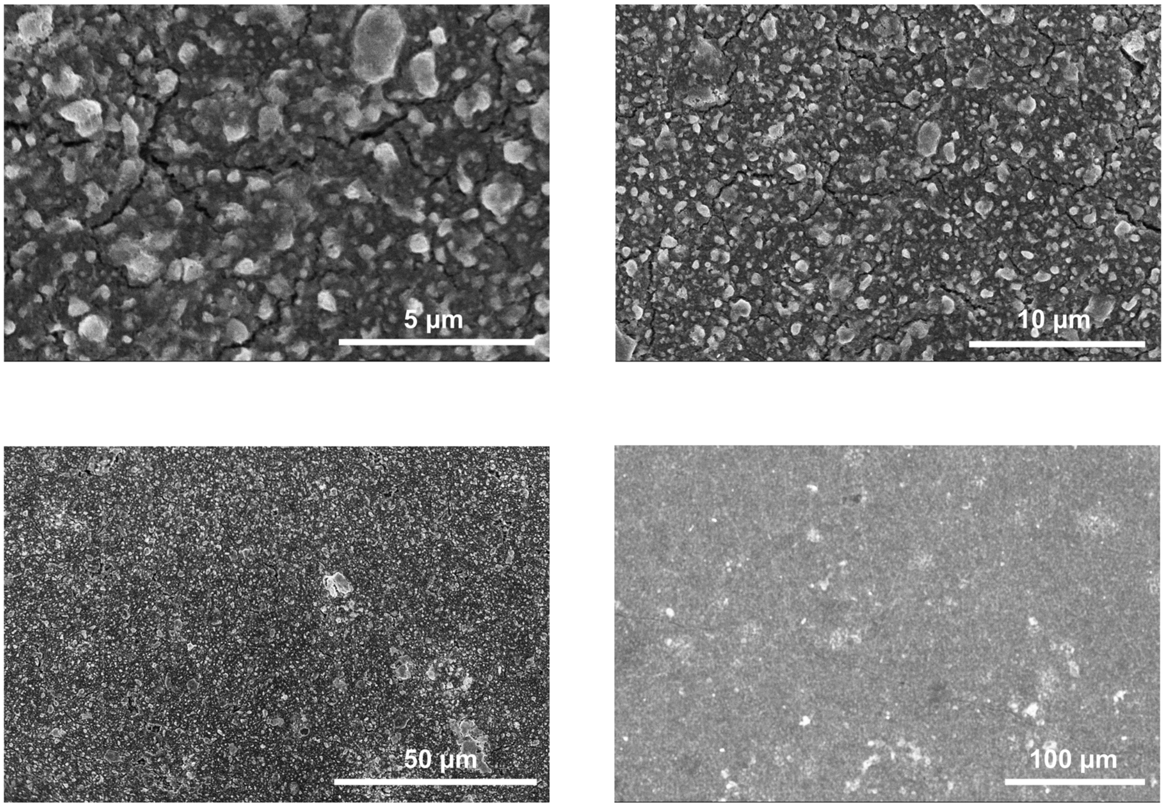

Supplementary Figure 7. Top view SEM images of the S composite electrode without LiI pelletized at 350MPa under different magnification.

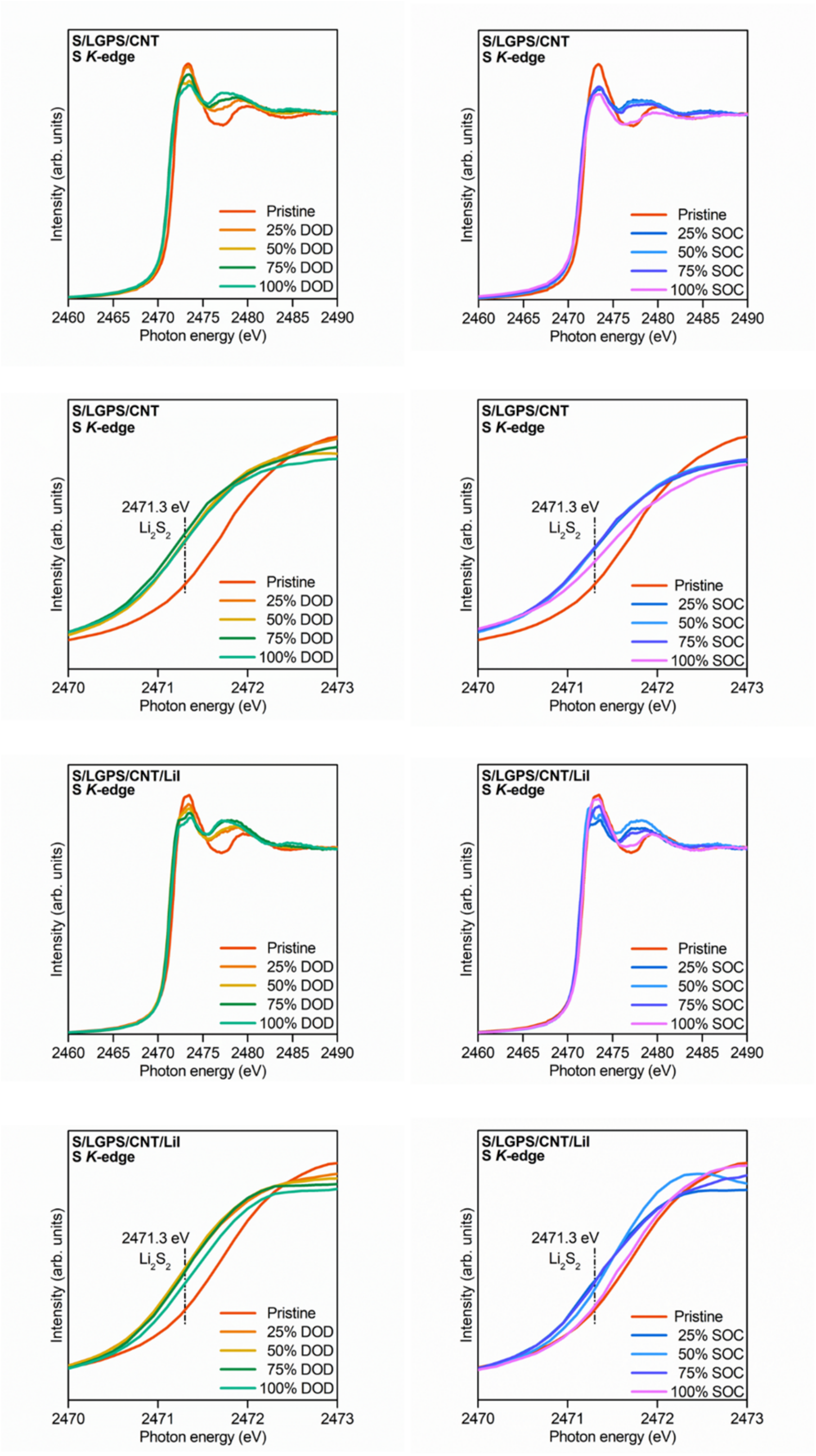
S/LGPS/CNT
S K-edge
Intensity (arb. units)
Photon energy (eV)
2460 2465 2470 2475 2480 2485 2490
Pristine
25% DOD
50% DOD
75% DOD
100% DOD
25% SOC
50% SOC
75% SOC
100% SOC
2470 2471 2472 2473
2471.3 eV
Li_2S_2
S/LGPS/CNT/LiI

Supplementary Figure 8. XANES spectra of ASSLSBs with and without LiI at different discharge/charge states.

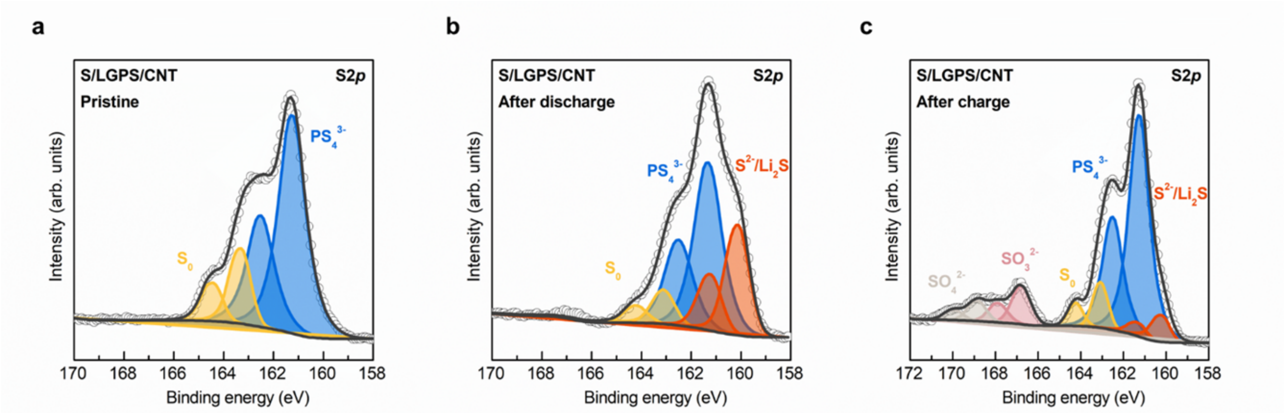

Supplementary Figure 9. S 2p XPS Spectra of ASSLSBs (a) before cycling, (b) after full discharge, and (c) after full charge. The S $2p_{3/2}$ binding energies of S_0, PS_4^{3-}, and S^{2-}/Li_2S, are 163.3 eV, 161.3 eV, and 1601.1 eV, respectively.

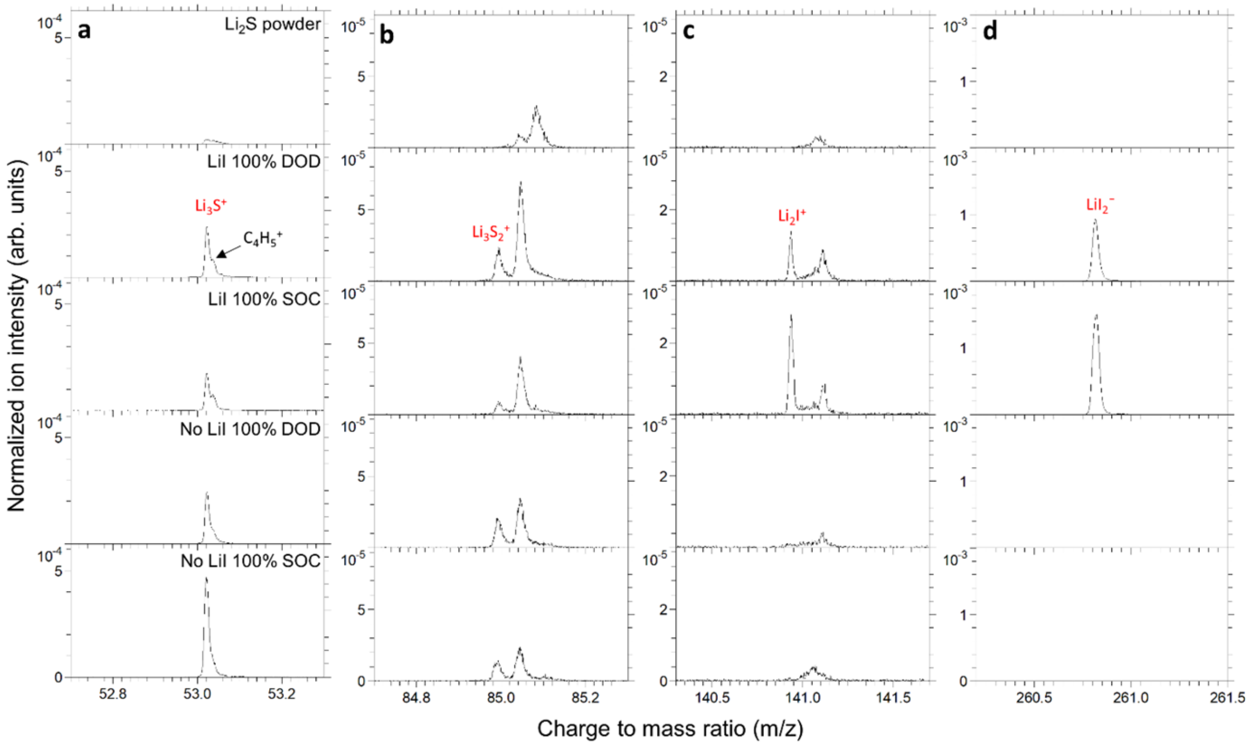

Supplementary Figure 10. ToF-SIMS secondary ion mass spectra in the mass ranges for ions (a) Li_3S^+, (b) $Li_3S_2^+$, (c) Li_2I^+ and (d) LiI_2^- for fully discharged (100% DOD) and

fully charged (100% SOC) products of ASSLSBs with and without LiI. The spectra are normalized to their total ion intensity for comparison purposes.

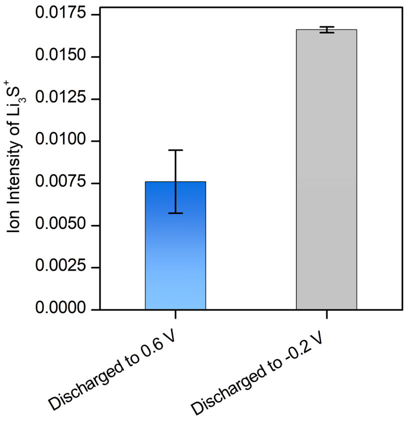

Supplementary Figure 11. Ion intensity of Li_3S^+ for ASSLSBs discharged to 0.6 V (vs. Li-In/Li^+) and 0.2 V (vs. Li-In/Li^+). The Li_3S^+ ion intensity is normalized to the total ion intensity. The error bars represent the standard deviation of the measured intensity ratio and were produced using five independent measurements.

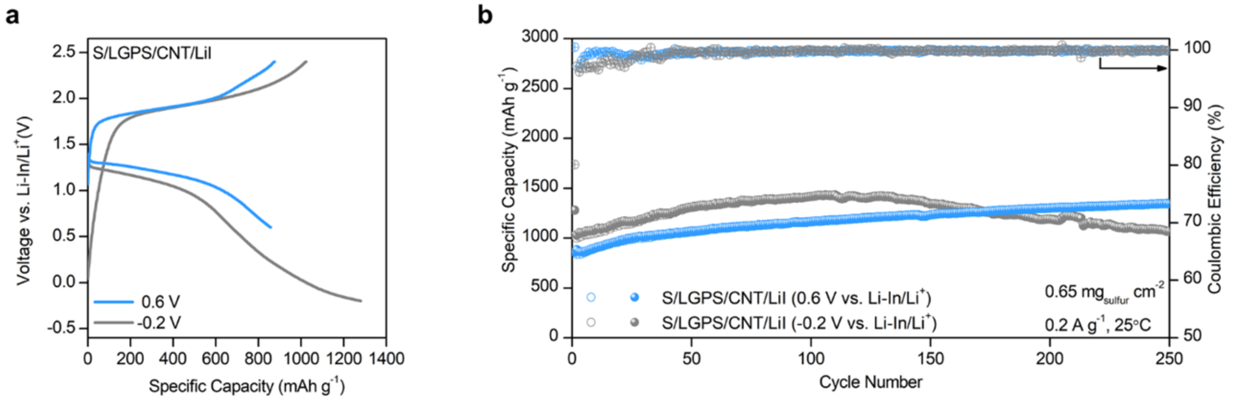

Supplementary Figure 12. (a) Voltage profile of ASSLSBs tested using different lower limit potentials. (b) Corresponding cycling performance of ASSLSBs tested at 0.2 A g^{-1} and 25°C.

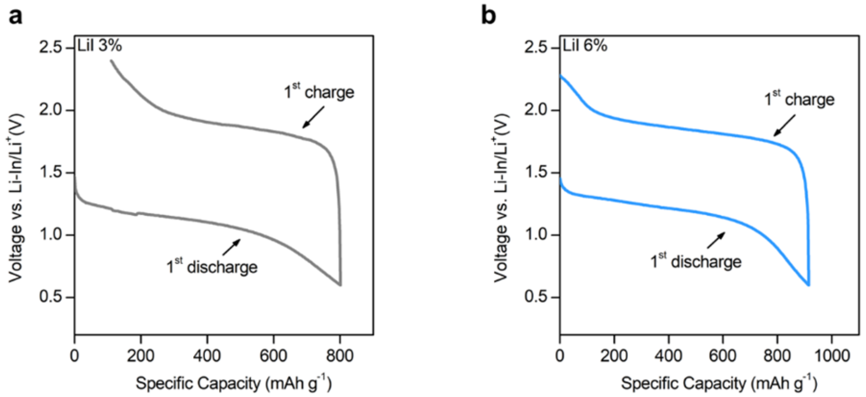

Supplementary Figure 13. (a) Voltage profile showing the reversibility of ASSLSBs in the first cycle with 3 wt% LiI. (b) Voltage profile showing the reversibility of ASSLSBs in the first cycle with 6 wt% LiI.

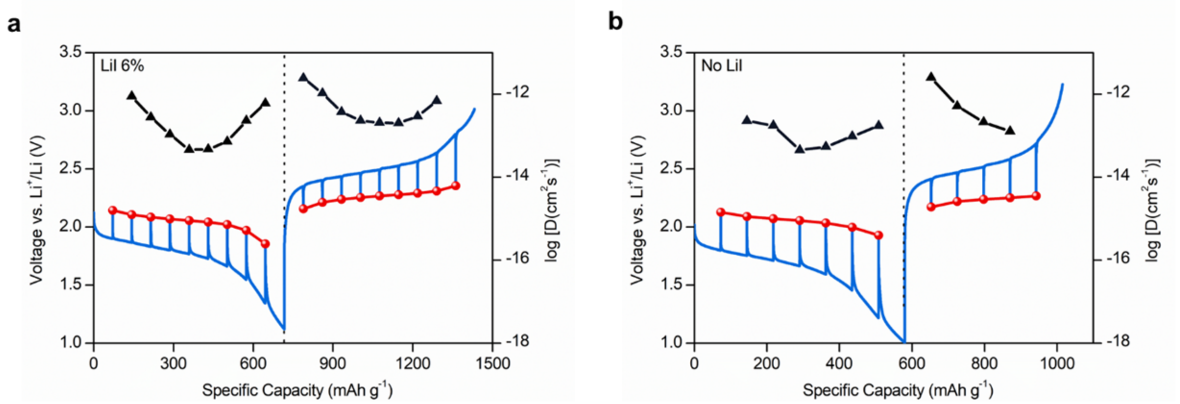

Supplementary Figure 14. GITT curve of ASSLSBs with and without LiI in the first cycle. 0.2 A g^{-1} current pulses are used for 20-minute increments followed by a 2 h relaxation.

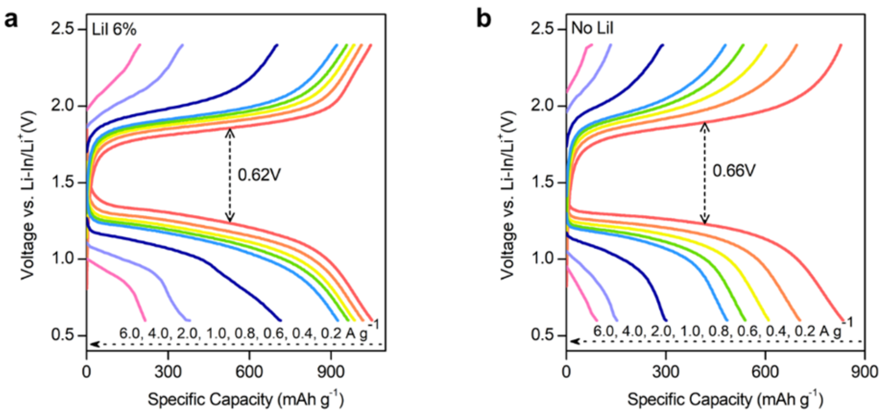

Supplementary Figure 15. Voltage profiles of ASSLSBs with and without LiI cycled over a current density range of 0.2 to 6.0 A g^{-1}.

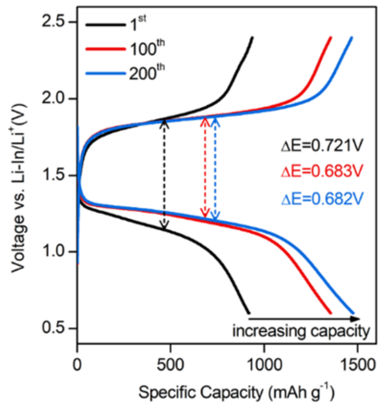

Supplementary Figure 16. Voltage profile of ASSLSB at 1st, 100th, and 200th cycle.

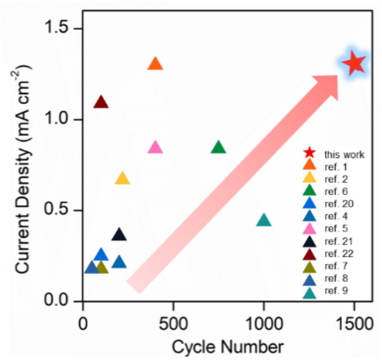

Supplementary Figure 17. Plot depicting the cycling behavior demonstrated in this work compared to recent ASSLSB reports.

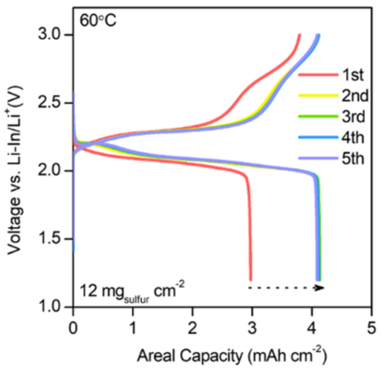

Supplementary Figure 18. Voltage profile of ASSLSBs with active material loading of 12 mg cm^{-2}.

Supplementary Note 1

Density Functional Theory (DFT) Calculations

The formation energies of Li_2S_2, Li_2S and S were calculated in vacuum and on the LiI surface to provide atomic level insights regarding the catalytic mechanism of LiI. An energy correction of -0.503 eV/atom for anionic sulfur in the structure was utilized based on a previous study conducted by Persson et al.[10] The calculated formation energy values for Li_2S_2, Li_2S and S in vacuum are in close agreement with the data available in the Materials Project database.[11] A slight difference was observed due to the use of GGA in this work, as compared to a mixture of GGA and GGA+U in the Materials Project Database for modelling exchange-correlation.[12] The calculated values for formation energies in vacuum are also in good agreement with a previous study conducted by Wang et al.[13] In vacuum, Li_2S has a formation energy of approximately -1.59 eV/atom and is shown to exist in the bulk phase rather than the molecular phase. This indicates that Li_2S is very stable and requires a large activation potential to facilitate its electrochemical oxidation back to S during charge. The calculated formation energy of Li_2S_2 and Li_2S on

the LiI(100) surface suggests that the compounds are thermodynamically stable in their molecular form on LiI(100), as compared to the compounds in vacuum. In contrast, the formation energy of S_8 on the LiI(100) surface is calculated to be approximately +0.02 eV/atom. This indicates that the S_8 molecule breaks off to form a bulk sulfur phase after it has been oxidized from Li_2S to S on the LiI(100) surface. These results demonstrate that LiI can facilitate the electrochemical oxidation of Li_2S_2 and Li_2S during charge.

To understand the delithiation process of Li_2S_2 and Li_2S during charging, the energy required to extract one lithium atom from bulk Li_2S and adsorbed Li_2S_2 and Li_2S was computed using DFT. Barrier to Li removal could not be calculated for bulk Li_2S_2 because it is meta-stable (energy above convex hull = 0.315 eV/atom), resulting in immediate structural decomposition.[14] For the adsorbed substrate, one Li atom was removed from the adsorbed Li_2S and Li_2S_2 molecule as per the following reaction: (1) $Li_2S \rightarrow$ Li-S* + Li* and (2) $Li_2S_2 \rightarrow$ Li-S-S* + Li*, respectively. The lithium extraction energy for the adsorbed Li_2S_2 and Li_2S molecules were calculated using the following expression: $E_{ext} = E(\text{Li-S*/ Li-S-S*}) - E(Li_2S_{(ads)}/\ Li_2S_{2(ads)})$. The lithium vacancy formation energy of a bulk Li_2S crystal was calculated using a large supercell of 96 atoms for bulk Li_2S, using the following expression: $Evac = E(Li_2S_{(\text{bulk with 1 Li vacancy})}) - E(Li_2S_{(\text{bulk})})$. The lithium extraction energy was calculated to be +4.10 eV and +3.78 eV for the adsorbed Li_2S and Li_2S_2 molecule, respectively, while the lithium vacancy formation energy of the bulk Li_2S crystal was calculated to be +5.75 eV per Li atom. After accounting for the energy required to form Li_2S on the LiI surface, the overall activation peak was lowered by 0.65 eV/atom, which indicates that the energy required for delithiation of Li_2S becomes lower in the

presence of LiI as compared to bulk Li_2S. On LiI(100) surface, the overall activation peak for Li_2S_2 as compared to Li_2S was 0.42 eV/atom lower.

Supplementary Note 2

Structural Characterization of Cathode Composites With and Without LiI

Two cathode composites with and without LiI were prepared by mixing sulfur (S), $Li_{10}GeP_2S_{12}$ (LGPS) SSE, carbon nanotubes (CNTs), and lithium iodide (LiI) using a ball-milling method. The absence of diffraction peaks in the XRD patterns for both the cathode composites with and without LiI indicates that the incorporated materials were homogeneously mixed after the ball-milling process (Supplementary Fig. 1a). The Raman spectra in Supplementary Fig. 1b shows that the peak at 472 cm^{-1} representative of the S-S bonds in elemental sulfur shows a slight redshift for both the composites with and without LiI. This implies a reduced interaction between S-S bonds, which is most likely caused by the CNTs and/or LiI.[15] The sulfur content in the cathode composites with and without LiI was approximately 30 and 36 wt%, respectively, as determined by thermogravimetric analysis (TGA) (Supplementary Fig. 1c). Interestingly, the sublimation temperature of sulfur in both cathodes slightly accelerates, in comparison to pure sulfur. This can be attributed to the high thermal conductivity of the CNTs, which accelerates sulfur sublimation during heating.[16] As LiI exhibits a lower thermal conductivity when compared to carbon, the sulfur in the LiI-incorporated cathode composite begins to sublime at a slightly higher temperature than its counterpart. The morphology of the ball-milled cathode composites with and without LiI are shown by the scanning electron microscopy (SEM) images in Supplementary Fig. 1d, Supplementary Fig. 2, and

Supplementary Fig. 3. Energy dispersive X-ray spectroscopy (EDX) confirms a homogenous distribution of S, Ge, P throughout both cathode composites and I for the composite with LiI (Supplementary Fig. 1d and Supplementary Fig. 4). The cross-sectional SEM image confirms that the LiI-incorporated cathode composite establishes good contact with the SSE interlayer (Supplementary Fig. 1e). Iodine ions have a larger ionic radius (r_{I^-} = 206 pm) compared to sulfur ions ($R_S{}^{2-}$ = 170 ppm), which means that Li-I has a longer ionic bond length and higher polarizability, indicating high Li^+ mobility and soft mechanical properties.[15,17] Top view SEM images of the pelletized cathode composites were taken to provide information about the surface morphology of the samples. The pellet with LiI exhibits a dense and compact morphology, indicating intimate contact between particles (Supplementary Fig. 1f and Supplementary Fig. 5). EDX mapping confirms the even distribution of elements (Supplementary Fig. 6). In contrast, the pellet without LiI has several cracks on the surface (Supplementary Fig. 1g and Supplementary Fig. 7). It can be inferred that LiI can improve contact at the tri-phase interface between sulfur, LGPS, and CNTs in the cathode composite. This phenomenon is consistent with other studies that have incorporated halide materials such as fluorine.[18,19]

References

1. Sakuda, A., Sato, Y., Hayashi, A. & Tatsumisago, M. Sulfur-Based Composite Electrode with Interconnected Mesoporous Carbon for All-Solid-State Lithium–Sulfur Batteries. *Energy Technol* **7**, 1900077 (2019).

2. Sun, X. *et al.* High Surface Area N-Doped Carbon Fibers with Accessible Reaction Sites for All-Solid-State Lithium-Sulfur Batteries. *Small* **18**, 2105678 (2022).

3. Han, Q. *et al.* Outstanding cycle stability and rate capabilities of the all-solid-state Li-S battery with a Li 7 P 3 S 11 glass-ceramic electrolyte and a core-shell S@BP2000 nanocomposite. *J Mater Chem A Mater* **7**, 3895–3902 (2019).

4. Hou, L.-P. *et al.* Improved interfacial electronic contacts powering high sulfur utilization in all-solid-state lithium–sulfur batteries. *Energy Storage Mater* **25**, 436–442 (2020).

5. Zhang, Q. *et al.* CNTs@S composite as cathode for all-solid-state lithium-sulfur batteries with ultralong cycle life. *J Energy Chem* **40**, 151–155 (2020).

6. Yao, X. *et al.* High-Performance All-Solid-State Lithium–Sulfur Batteries Enabled by Amorphous Sulfur-Coated Reduced Graphene Oxide Cathodes. *Adv Energy Mater* **7**, (2017).

7. Zhu, G. L. *et al.* A Self-Limited Free-Standing Sulfide Electrolyte Thin Film for All-Solid-State Lithium Metal Batteries. *Adv Funct Mater* **31**, (2021).

8. Wang, S. *et al.* High-Conductivity Argyrodite Li 6 PS 5 Cl Solid Electrolytes Prepared via Optimized Sintering Processes for All-Solid-State Lithium-Sulfur Batteries. *ACS Appl Mater Interfaces* **10**, 42279–42285 (2018).

9. Zhang, Y. *et al.* High-performance all-solid-state lithium-sulfur batteries with sulfur/carbon nano-hybrids in a composite cathode. *J Mater Chem A Mater* **6**, 23345–23356 (2018).

10. Wang, A. *et al.* A framework for quantifying uncertainty in DFT energy corrections. *Sci Rep* **11**, 15496 (2021).

11. Jain, A. *et al.* Commentary: The Materials Project: A materials genome approach to accelerating materials innovation. *APL Mater* **1**, 011002 (2013).

12. Jain, A. *et al.* Formation enthalpies by mixing GGA and GGA <math display="inline"> <mrow> <mo>+</mo> </mrow> </math> <math display="inline"> <mrow> <mi>U</mi> </mrow> </math> calculations. *Phys Rev B* **84**, 045115 (2011).

13. Gao, G., Zheng, F., Pan, F. & Wang, L. Theoretical Investigation of 2D Conductive Microporous Coordination Polymers as Li–S Battery Cathode with Ultrahigh Energy Density. *Adv Energy Mater* **8**, 1801823 (2018).

14. The Materials Project. *Materials Data on LiS by Materials Project*. United States: N. p., 2020. Web. doi:10.17188/1666639.

15. Wan, H. *et al.* Understanding LiI-LiBr Catalyst Activity for Solid State Li_2 S/S Reactions in an All-Solid-State Lithium Battery. *Nano Lett* **21**, 8488–8494 (2021).

16. Kumanek, B. & Janas, D. Thermal conductivity of carbon nanotube networks: a review. *J Mater Sci* **54**, 7397–7427 (2019).

17. Asano, T. *et al.* Solid Halide Electrolytes with High Lithium-Ion Conductivity for Application in 4 V Class Bulk-Type All-Solid-State Batteries. *Adv Mater* **30**, 1803075 (2018).

18. Zhang, S. *et al.* Advanced High-Voltage All-Solid-State Li-Ion Batteries Enabled by a Dual-Halogen Solid Electrolyte. *Adv Energy Mater* **11**, 2100836 (2021).

19. Mo, F. *et al.* Inside or Outside: Origin of Lithium Dendrite Formation of All Solid-State Electrolytes. *Adv Energy Mater* **9**, 1902123 (2019).

20. Alzahrani, A. S. *et al.* Confining Sulfur in Porous Carbon by Vapor Deposition to Achieve High-Performance Cathode for All-Solid-State Lithium–Sulfur Batteries. *ACS Energy Lett* **6**, 413–418 (2021).

21. Pan, H. *et al. Carbon-free and binder-free Li-Al alloy anode enabling an all-solid-state Li-S battery with high energy and stability*. *Sci. Adv* **8**, eabn4372 (2022).

22. Zhu, X. *et al.* Exploring the concordant solid-state electrolytes for all-solid-state lithium-sulfur batteries. *Nano Energy* **96**, (2022).
